# Timing and Predictors of Loss of Infectivity Among Healthcare Workers With Mild Primary and Recurrent COVID-19: A Prospective Observational Cohort Study

**DOI:** 10.1093/cid/ciad535

**Published:** 2023-09-07

**Authors:** Stefania Dzieciolowska, Hugues Charest, Tonya Roy, Judith Fafard, Sara Carazo, Ines Levade, Jean Longtin, Leighanne Parkes, Sylvie Nancy Beaulac, Jasmin Villeneuve, Patrice Savard, Jacques Corbeil, Gaston De Serres, Yves Longtin

**Affiliations:** McGill University Faculty of Medicine, Montréal, Canada; Faculté de médecine, Université de Montréal, Montréal, Canada; Laboratoire de Santé Publique du Québec, Sainte-Anne-de-Bellevue, Canada; Institut National de Santé Publique du Québec, Québec City, Canada; Laboratoire de Santé Publique du Québec, Sainte-Anne-de-Bellevue, Canada; Institut National de Santé Publique du Québec, Québec City, Canada; Laboratoire de Santé Publique du Québec, Sainte-Anne-de-Bellevue, Canada; Institut National de Santé Publique du Québec, Québec City, Canada; Institut National de Santé Publique du Québec, Québec City, Canada; Université Laval, Québec City, Canada; Laboratoire de Santé Publique du Québec, Sainte-Anne-de-Bellevue, Canada; Institut National de Santé Publique du Québec, Québec City, Canada; CHU de Québec—Université Laval, Québec City, Canada; McGill University Faculty of Medicine, Montréal, Canada; Jewish General Hospital Sir Mortimer B. Davis, Montréal, Canada; Laboratoire de Santé Publique du Québec, Sainte-Anne-de-Bellevue, Canada; Institut National de Santé Publique du Québec, Québec City, Canada; Institut National de Santé Publique du Québec, Québec City, Canada; Faculté de médecine, Université de Montréal, Montréal, Canada; Centre Hospitalier de l’Université de Montréal (CHUM) and CHUM Research Center, Montréal, Canada; Université Laval, Québec City, Canada; Institut National de Santé Publique du Québec, Québec City, Canada; Université Laval, Québec City, Canada; McGill University Faculty of Medicine, Montréal, Canada; Jewish General Hospital Sir Mortimer B. Davis, Montréal, Canada; Lady Davis Research Institute, Montréal, Canada

**Keywords:** SARS-CoV-2, COVID-19, infectivity, recurrent infection, viral culture

## Abstract

**Background:**

There is a need to understand the duration of infectivity of primary and recurrent coronavirus disease 2019 (COVID-19) and identify predictors of loss of infectivity.

**Methods:**

Prospective observational cohort study with serial viral culture, rapid antigen detection test (RADT) and reverse transcription polymerase chain reaction (RT-PCR) on nasopharyngeal specimens of healthcare workers with COVID-19. The primary outcome was viral culture positivity as indicative of infectivity. Predictors of loss of infectivity were determined using multivariate regression model. The performance of the US Centers for Disease Control and Prevention (CDC) criteria (fever resolution, symptom improvement, and negative RADT) to predict loss of infectivity was also investigated.

**Results:**

In total, 121 participants (91 female [79.3%]; average age, 40 years) were enrolled. Most (n = 107, 88.4%) had received ≥3 severe acute respiratory syndrome coronavirus 2 (SARS-CoV-2) vaccine doses, and 20 (16.5%) had COVID-19 previously. Viral culture positivity decreased from 71.9% (87/121) on day 5 of infection to 18.2% (22/121) on day 10. Participants with recurrent COVID-19 had a lower likelihood of infectivity than those with primary COVID-19 at each follow-up (day 5 odds ratio [OR], 0.14; *P* < .001]; day 7 OR, 0.04; *P* = .003]) and were all non-infective by day 10 (*P* = .02). Independent predictors of infectivity included prior COVID-19 (adjusted OR [aOR] on day 5, 0.005; *P* = .003), an RT-PCR cycle threshold [Ct] value <23 (aOR on day 5, 22.75; *P* < .001) but not symptom improvement or RADT result.

The CDC criteria would identify 36% (24/67) of all non-infectious individuals on day 7. However, 17% (5/29) of those meeting all the criteria had a positive viral culture.

**Conclusions:**

Infectivity of recurrent COVID-19 is shorter than primary infections. Loss of infectivity algorithms could be optimized.

## Teaser

Among 121 individuals with COVID-19, viral culture positivity decreased from 72% on day 5 of infection to 18% on day 10. Predictors of loss of infectivity included a history of previous infection and a RT-PCR cycle threshold value >27.

## Graphical Abstract

**Graphical Abstract:**
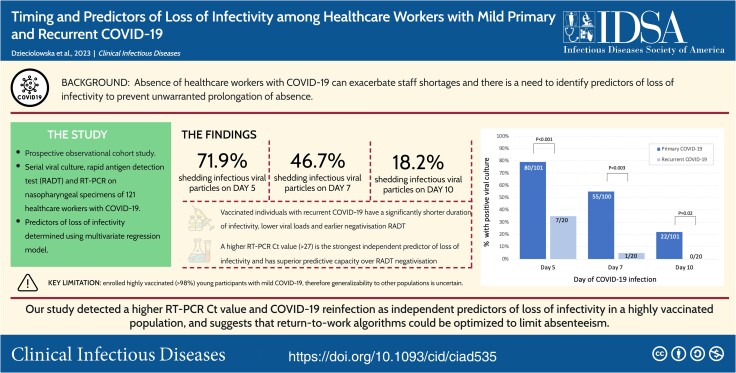
This graphical abstract is also available at Tidbit: https://tidbitapp.io/tidbits/timing-and-predictors-of-loss-of-infectivity-among-healthcare-workers-with-mild-primary-and-recurrent-covid-19-a-prospective-observational-cohort-study-9a1bfc5d-3c21-4659-a057-701bb1b5b542

Coronavirus disease 2019 (COVID-19) is caused by the severe acute respiratory syndrome coronavirus 2 (SARS-CoV-2) [1]. The current evidence regarding duration of infectivity rely on viral culture to detect shedding of replication-competent virus (also called viable or infectious virus). These studies suggest that immunocompetent individuals with non-severe COVID-19 can remain infective for up to 10 days [2–6].

Although infective, healthcare workers (HCWs) must refrain from working to prevent nosocomial transmission [7, 8]. The timing of their return to work is complicated by the interindividual variation in the durations of infectivity. Approximately a fifth of individuals may be infective for as little as 5 days, whereas approximately a quarter can remain infective for ≥10 days [9]. Determinants of loss of infectivity are largely unknown, but could be useful to optimize return-to-work policies. To limit absenteeism [10], the US Centers for Disease Control and Prevention (CDC) and European CDC have provided guidance to allow earlier return to work of eligible HCWs using information such as symptom improvement and the result of rapid antigen detection tests (RADT) [7, 8]. However, whether these criteria can reliably distinguish infective and non-infective individuals remain unclear.

Furthermore, many studies that investigated duration of infectivity were conducted early in the pandemic when individuals were infected for the first time and often were unvaccinated. Few studies have investigated duration of infectivity of recurrent COVID-19 [9]. Hence, we sought to evaluate the duration of infectivity of HCWs infected with primary and recurrent COVID-19, and identify predictors of infectivity using viral culture as a marker of infectivity.

## METHODS

### Study Population

We conducted a prospective observational cohort study between 20 February 2022 and 6 March 2023 at a large healthcare organization employing 12 000 HCWs across 20 institutions. Participants were identified through the Occupational Health service. Inclusion criteria included (1) SARS-CoV-2 infection confirmed by reverse transcription polymerase chain reaction (RT-PCR) from a nasopharyngeal specimen, and (2) symptom onset <72 hours prior to enrolment. Exclusion criteria included asymptomatic infections; moderate-to-severe COVID-19 (World Health Organization [WHO] Ordinal Scale for Clinical Improvement ≥3) [11]; contraindication to nasopharyngeal sampling; and use of COVID-19-specific therapies (eg antivirals). Participants were followed on the fifth, seventh, and tenth day of their infection (with the day of onset of symptoms defined as day 1).

The study follows the Strengthening the Reporting of Observational Studies in Epidemiology (STROBE) guideline [12] and was approved by local research ethics committees (2022–3235). Written informed consent was obtained from participants.

### Data Collection

Clinical data included sociodemographic information, medical history (including prior laboratory-confirmed COVID-19), COVID-19 vaccination status (including number of doses and manufacturer), and symptomatology. We also assessed the use of antipyretics (acetaminophen and non-steroidal anti-inflammatory drugs) among afebrile participants as their use can mask fever. Participants reported this information online (LimeSurvey, Hamburg, Germany).

### Outcome Definitions

SARS-CoV-2 infectivity was defined as evidence of cytopathic effect (CPE) on microscopy of viral culture from a nasopharyngeal specimen, with etiology of the CPE being confirmed as SARS-CoV-2 by RT-PCR on the culture supernatant demonstrating at least 3 cycle threshold (Ct) values lower than the RT-PCR on the original sample [13]. Duration of infectivity was defined as the number of days between the onset of symptoms and the last positive culture.

### Laboratory Methods

Nasopharyngeal samples using a flocked swab (FLOQSwabs, Copan Italia) were placed in 3 mL universal viral transport media (UTM, Copan Italia) and kept at −80°C. Primary samples and supernatants were processed with an in-house RT-PCR targeting the SARS-CoV-2 N gene as previously described [14]. Forward, reverse, and probe sequences were as follows: AACCAGAATGGAGAACGCAGTG, CGGTGAACCAAGACGCAGTATTAT, and CGATCAAAACAACGTCGGCCCCAAGGTTTAC [14].

Viral cultures were performed on Vero E6 cells by blinded technologists as previously described using 0.1 mL of specimen as inoculum [14]. This cell line is commonly used to cultivate SARS-CoV-2 and has a median tissue culture infectious dose (TCID_50_) ranging between 2.0E+04 and 6.3E+06 [15]. Cultures were incubated at 35°C–37°C in 5% CO_2_ for 15 days.

All initial samples were sequenced to determine SARS-CoV-2 lineage using the Illumina technology. Data analysis was performed using the GenPipes Covseq pipeline [16], and variant identification was performed with Pangolin program (see appendix for details) [17].

Lateral-flow RADT were provided to participants (COVID-19 Antigen Rapid Test, BTNX, Hannover, Germany) who performed the tests on self-sampled midturbinate swab specimens by following the manufacturer's instructions [18].

### Sample Size Estimate

Based on studies indicating that 25% of individuals remain infective on the seventh day of their infection [19], we estimated that recruiting 120 participants would provide a precision of ± 7% at the 95% confidence interval (CI).

### Statistical Analyses

Descriptive statistics reported discrete variables as numbers and proportions, and continuous variables as mean ± standard deviation (SD) or median and interquartile ranges (IQR). Ct values were categorized according to quartiles on day 5. The primary outcome was the proportion of HCWs with evidence of infectivity on the fifth, seventh, and tenth day of their infection.

To investigate the capacity of the RT-PCR Ct value (an indicator of viral load that is inversely proportional to the quantity of nucleic acid in a sample) and RADT to predict infectivity, the Ct values of samples with positive versus negative culture was depicted in the form of boxplots with overlaid jitter plot.

To investigate factors associated with persistent infectivity, odds ratios (OR) and 95% CI were estimated using bivariate and multivariate logistic regression at each visit. Multivariate models included clinical characteristics (symptom severity, symptom resolution, and fever) and results laboratory assays (RADT and RT-PCR) collected at each follow-up, as well as baseline individual (age, sex, and immunological status) and viral (SARS-CoV-2 lineage) information. These variables were pre-defined as potential predictors of infectivity according to literature and current practices [7, 8]. Categories were grouped when necessary for model convergence. Variables perfectly predicting the presence of infectivity could not be included in the corresponding multivariate model. Immunologic status was categorized according to vaccination and prior infection as follows: recent vaccination (last dose received <6 months ago) without prior infection; non-recent vaccination (last dose received ≥6 months ago) without prior infection; and hybrid immunity (vaccination at any time and prior infection).

### Performance of Return-to-Work Algorithms

We estimated the capacity of the CDC algorithm to discriminate infective and non-infective HCWs on day 7 of their infection [7]. We also quantified the probability of an infectious HCW returning to work and estimated the impact of these criteria on absenteeism. Finally, we explored the performance of alternative algorithms that were derived from variables identified in the current study. These evaluations assumed that in the absence of return-to-work criteria, HCWs would return to work 10 days after the onset of their symptoms.

All analyses were performed in SAS version 9.4. All tests were 2-tailed, and a *P* value < .05 was considered statistically significant. Adjustment for multiple comparisons were not applied in this exploratory study [20, 21].

### Role of Funding Source

The study was sponsored by the Ministère de la Santé et des Services Sociaux du Québec and the Public Health Agency of Canada. The sponsors had no role in the design, conduct and reporting of the study.

## RESULTS

**Figure 1.**
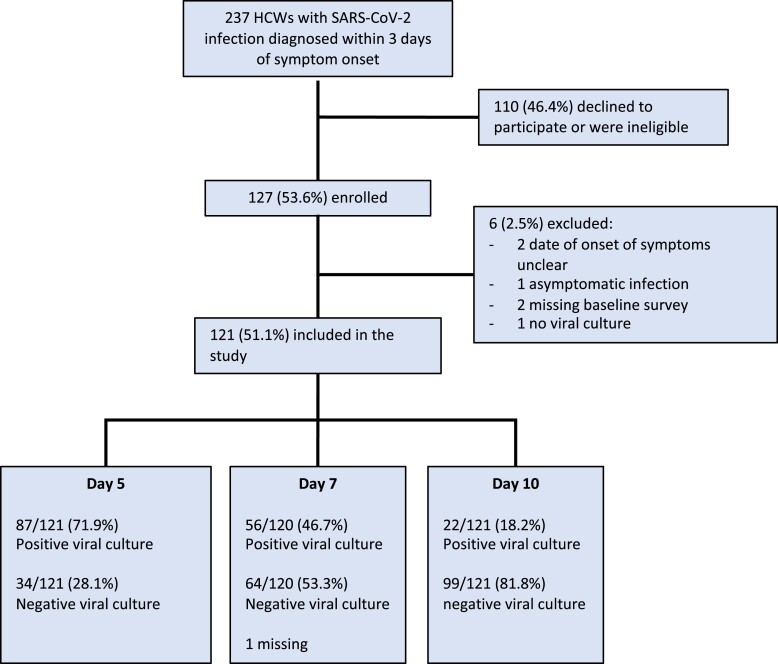
Flow diagram of participant selection into the study and proportion of infective participants at each follow-up visit. Abbreviations: HCW, healthcare worker; SARS-CoV-2, severe acute respiratory syndrome coronavirus 2.

Overall, 121 (51.1%) participants were included in the analyses (Figure 1), and 714 specimens (360 nasopharyngeal and 354 mid-turbinate swabs) were collected. Characteristics of participants are shown in Table 1; 79.3% (96/121) were female, and the average age was 40.2 (SD, 12.0 years). The infections were due to multiple Omicron lineages including BA.1 (11.6%), BA.2 (60.3%), and BA.5 (8.3%), inclusive of sublineages. Virtually all participants (98.3%) were previously immunized with ≥ 1 dose of SARS-CoV-2 vaccine. Most (84.3%) had received 3 doses, most commonly the Pfizer–BioNTech Comirnaty (89.3% of all received doses). The median elapsed time between the last dose and the current infection was 122 days (IQR, 95–175 days). Twenty (20) participants (16.5%) had a prior COVID-19 episode. All these previous episodes were mild (WHO Grade scale ≤2) and occurred a median of 347.5 days prior to the current episode (IQR, 264 to 454 days).

**Table 1.** Demographic and Clinical Characteristics of Healthcare Workers With COVID-19

Characteristic	Overall Population (n = 121)
Demographic characteristics	
Mean age—y (SD)	40.2 (12.0)
Female sex (%)	96 (79.3)
Workplace	
Acute care hospital (%)	56 (46.3)
Local community services centers (%)	16 (13.2)
Long term care facilities (%)	15 (12.4)
Rehabilitation center (%)	9 (7.4)
Private clinic, family medicine clinic (%)	7 (5.8)
Other^a^ (%)	18 (14.9)
Occupation	
Nurse, nurse practitioner, patient care attendant (%)	45 (37.2)
Physician (%)	20 (16.5)
Administration (%)	13 (10.7)
Physiotherapy, occupational therapy, social worker, radiology technician (%)	22 (18.2)
Other (%)	21 (17.4)
Comorbidities and past medical history	
Immunocopromised condition^b^ (%)	4 (3.3)
Previous COVID-19 episode (%)	20 (16.5)
Median elapsed time since last COVID-19 episode—d (IQR)	347.5 (264–454)
COVID-19 vaccination status	
Not vaccinated (%)	2 (1.7)
1 dose (%)	3 (2.5)
2 doses (%)	9 (7.4)
3 doses (%)	102 (84.3)
4 doses (%)	5 (4.1)
COVID-19 vaccine type (n = 347 doses)^d^	
Pfizer-BioNTech Comirnaty (%)	310 (89.3)
Moderna Spikevax (%)	30 (8.6)
AstraZeneca Vaxzevria (%)	7 (2.0)
Median elapsed time since last COVID-19 vaccine dose—d (IQR)	122 (95–175)
Severity of COVID-19 infection^c^	
Very mild (Ambulatory, no limitation of activities) (%)	97 (80.2)
Mild (Ambulatory, with limitation of activities) (%)	24 (19.8)
SARS-CoV-2 specific therapy^e^ (%)	1 (0.8)
COVID-19 symptomatology on enrollment	
Median number of symptoms (IQR)	5 (3–6)
Sore throat (%)	94 (77.7)
Rhinorrhea and/or nasal congestion (%)	88 (72.7)
Fatigue (%)	81 (66.9)
Headache (%)	77 (63.6)
Myalgia (%)	55 (45.5)
Chills (%)	50 (41.3)
Cough (%)	21 (17.4)
Fever (%)	18 (14.9)
Dizziness (%)	17 (14.0)
Diarrhea (%)	14 (11.6)
Nausea and/or vomiting (%)	10 (8.3)
Chest pain (%)	10 (8.3)
Dyspnea (%)	8 (6.6)
Anosmia (%)	3 (2.5)
Ageusia (%)	3 (2.5)
SARS-CoV-2 lineage	
BA.1 and sublineages (%)	14 (11.6)
BA.2 and sublineages (%)	73 (60.3)
BA.4 and sublineages (%)	3 (2.5)
BA.5 (%)	10 (8.3)
BQ.1 (%)	9 (7.4)
XBB (%)	1 (0.8)
Recombinants (%)	2 (1.7)
Unknown (%)	9 (7.4)

Abbreviations: COVID-19, coronavirus disease 2019; IQR, interquartile range; SARS-CoV-2, severe acute respiratory syndrome coronavirus 2; SD, standard deviation.

^a^Includes vaccination center (n = 3), research institute (n = 5), rehabilitation centers (n = 3), health phone services (n = 5), cancer wellness center (n = 1), and medical school (n = 1).

^b^Includes multiple sclerosis receiving fingolimod (n = 1), multiple myeloma post autologous stem cell transplantation (n = 1), colorectal cancer under chemotherapy (n = 1), and Crohn disease receiving anti-tumor necrosis factor (anti-TNF) therapy.

^c^Severity determined by the World Health Organization Ordinal Scale for Clinical Improvement.

^d^Sum of percentages is greater than 100% because of rounding.

^e^A single patient received nirmatrelvir and ritonavir after enrollment into the study.

### Symptoms of Current COVID-19

Upon enrollment, all participants described their infection as “very mild” or “mild” (WHO Grade scale of 1 or 2). The most common symptoms were sore throat (77.7%), rhinorrhea and/or nasal congestion (72.7%) and fatigue (66.9%). The clinical evolution was favourable. No participant was hospitalized. A single participant received nirmatrelvir-ritonavir after enrollment. The proportion of participants with markedly improved or resolved symptoms increased from 43.8% on day 5% to 84.3% on day 10 (data not shown). Fever was uncommon: only 14.9% were febrile on enrollment. However, antipyretics were used by 50% and 31% of afebrile individuals on day 5 and 7 of their infection, respectively.

### Evolution of Infectivity and Viral Shedding

The proportion of participants with a positive viral culture was 71.9% (87/121; 95% CI, 63.0% to 79.7%) on day 5, 46.7% (56/120; 95% CI, 37.5% to 56.0%) on day 7, and 18.2% (22/121; 95% CI, 11.8% to 26.2%) on day 10, respectively (Figure 1). The proportion of participants with a positive RT-PCR decreased from 93.3% (112/120) on day 5 (median Ct value, 23.4 [IQR, 20.6–27.9]) to 61.2% (74/120) on day 10 (median Ct value, 32.5 [IQR, 28.5 to undetectable]). Similarly, the proportion of RADT tests that were positive decreased from 81.5% (97/119) on day 5 to 34.2% (40/117) on day 10.

### Factors Associated With Infectivity

In bivariate analysis, multiple variables were associated with a positive viral culture (Table 2 and Supplementary Table 1 in supplementary appendix). A history of previous COVID-19 was strongly associated with a decreased likelihood of infectivity at each follow-up visit. Only 35% (7/20) of individuals with recurrent COVID-19 were still infective on day 5 compared with 79% of those with a primary episode (OR, 0.14; 95% CI .05–.40; *P* < .001). Similarly, only 5% (1/20) of participants with recurrent COVID-19 were still infective on day 7, compared with 55% (55/100) of those with a first episode (OR, 0.04; 95% CI, .01–.33; *P* = .003). Finally, the proportion of participants with primary versus recurrent infection that were still infective on day 10 were 22% versus 0%, respectively (*P* = .02 by Fisher exact test).

**Table 2.** Predictors of Infectivity on Day 5, 7, and 10 of COVID-19 Among Healthcare Workers (Bivariate Analyses)

	Day 5^b^				Day 7^b^				Day 10^b^			
Explanatory Variable	Absence of Infectivity n (Line %)	Presence of Infectivity n (Line %)	OR (95% CI)	*P* Value^c^	Absence of Infectivity n (Line %)	Presence of Infectivity n (Line %)	OR (95% CI)	*P* Value^c^	Absence of Infectivity n (Line %)	Presence of Infectivity n (Line %)	OR (95% CI)	*P* Value^c^
Overall	34 (28.1)	87 (71.9)	…		64 (53.3)	56 (46.7)	…		99 (81.8)	22 (18.2)	…	
Demographics												
Median age (IQR)	40 (34–53)	38 (30–48)	NE	.12	38.5 (31.5–49)	39.5 (32–48)	NE	.99	38 (31–48)	39.5 (29–51)	NE	.84
Male sex (%)	7 (28.0)	18 (72.0)	Ref		13 (20.3)	11 (19.6)	Ref		21 (84.0)	4 (16.0)	Ref	
Female sex (%)	27 (28.1)	69 (71.9)	0.99 (.37–2.65)	.99	51 (53.1)	45 (46.9)	1.04 (.43–2.56)	.93	78 (81.3)	18 (18.8)	1.21 (.37–3.96)	.75
Previous infection status												
No previous COVID-19	21 (20.8)	80 (79.2)	Ref		45 (45.0)	55 (55.0)	Ref		79 (78.2)	22 (21.8)	Ref	
Previous COVID-19	13 (65.0)	7 (35.0)	0.14 (.05–.40)	<.001	19 (95.0)	1 (5.0)	0.04 (.01–.33)	.003	20 (100)	0 (0.0)	NE	.02
Vaccination: number of doses received												
No vaccination or 1 dose received	2 (40.0)	3 (60.0)	Ref		2 (40.0)	3 (60.0)	Ref		5 (100)	0 (0.0)	Ref	
≥ 2 doses received	32 (27.6)	84 (72.4)	1.75 (.28–10.96)	.55	62 (53.9)	53 (46.1)	0.57 (.09–3.54)	0.55	94 (81.0)	22 (19.0)	NE	.58
Immunity status stratified by timing of last vaccine and previous COVID-19												
No previous infection and last vaccine dose ≥6 m ago	2 (16.7)	10 (83.3)	Ref		7 (63.6)	4 (36.4)	Ref		11 (91.7)	1 (8.3)	Ref	
No previous infection and last vaccine dose <6 m ago	19 (21.3)	70 (78.7)	0.74 (.15–3.65)	.71	38 (42.7)	51 (57.3)	2.35 (.64–8.60)	.20	68 (76.4)	21 (23.6)	3.40 (.41–27.87)	.26
Previous infection, last vaccine dose> or <6 m ago^a^	13 (65.0)	7 (35.0)	0.11 (.02–.64)	.01	19 (95.0)	1 (5.0)	0.09 (.01–.97)	.047	20 (100)	0 (0.0)	NE	.38
RADT result												
Negative	8 (61.5)	5 (38.5)	Ref		29 (85.3)	5 (14.7)	Ref		64 (100)	0 (0)	Ref	
Positive	20 (20.6)	77 (79.4)	6.16 (1.82–20.88)	.004	26 (34.7)	49 (65.3)	10.93 (3.78–31.60)	<.001	22 (55.0)	18 (45.0)	NE	.03
Uncertain	6 (66.7)	3 (33.3)	0.80 (.13–4.75)	.81	7 (87.5)	1 (12.5)	0.83 (.08–8.27)	.87	11 (84.6)	2 (15.4)	NE	<.001
SARS-CoV2 RT-PCR												
Median Ct value (IQR)	28.5 (25.0–33.4)	21.8 (20.3–25.0)	…	<.001	31.3 (27.4–35.6)	24.7 (22.9–27.4)	…	<.001	35.5 (31.4–40.0)	26.7 (24.4–28.3)	…	.002
Negative result	6 (75.0)	2 (25.0)	Ref		13 (100)	0 (0.0)	Ref		46 (100)	0 (0.0)	Ref	
Positive result	28 (25.0)	84 (75.0)	9.00 (1.72–47.17)	.01	51 (47.7)	56 (52.3)	NE	<.001	52 (70.3)	22 (29.7)	NE	<.001
RT-PCR Ct (reference: negative RT-PCR)												
Ct value: 27–34	15 (57.7)	11 (42.3)	2.20 (.37–13.04)	.39	36 (69.2)	16 (30.8)	Ref (Ct ≥27)	…	40 (81.6)	9 (18.4)	Ref (Ct ≥27)	…
Ct value: 23–<27	9 (33.3)	18 (66.7)	6.00 (1.00–35.91)	.05	13 (33.3)	26 (66.7)	6.12 (2.56–14.66)	<.001	10 (47.6)	11 (52.4)	9.67 (1.21–77.12)	.03
Ct value: 20–<23	3 (7.9)	35 (92.1)	35.00 (4.79–255.47)	.001	2 (15.4)	11 (84.6)	16.84 (3.37–84.17)	<.001	2 (50.0)	2 (50.0)	10.63 (3.55–31.86)	<.001
Ct value: <20	1 (4.8)	20 (95.2)	60.00 (4.60–782.36)	.002	0 (0.0)	3 (100)	NE	.02	0 (0.0)	0 (0.0)	NE	NE
SARS-CoV-2 lineage												
BA.2	21 (28.8)	52 (71.2)	Ref		39 (53.4)	34 (46.6)	Ref		62 (84.9)	11 (15.1)	Ref	
BA.1	0 (0.0)	14 (100.0)	NE	.02	1 (7.1)	13 (92.9)	14.91 (1.85–199.99)	.01	8 (57.1)	6 (42.9)	4.23 (1.23–14.57)	.02
BA.4/5	3 (23.1)	10 (76.9)	1.35 (.34–5.38)	.67	8 (66.7)	4 (33.3)	0.57 (.16–2.07)	.40	10 (76.9)	3 (23.1)	1.69 (.40–7.14)	.48
Others (BQ.1, XBB.1, recombinant, unknown)	10 (47.6)	11 (52.4)	0.44 (.16–1.20)	.11	16 (76.2)	5 (23.8)	0.36 (.12–1.08)	.07	19 (90.5)	2 (9.5)	.59 (.12–2.91)	.52
Severity of symptoms												
Asymptomatic	3 (60.0)	2 (40.0)	Ref		11 (57.9)	8 (42.1)	Ref		38 (88.4)	5 (11.6)	Ref	
Very mild^d^	28 (26.7)	77 (73.3)	4.12 (.65–25.99)	.13	50 (54.3)	42 (45.7)	1.16 (.43–3.14)	.78	58 (79.5)	15 (20.5)	1.97 (.66–5.86)	.23
Mild^d^	3 (33.3)	6 (66.7)	3.00 (.31–28.84)	.34	1 (16.7)	5 (83.3)	6.87 (.67–70.81)	.11	1 (100)	0 (0.0)	NE	1.000
Evolution of symptoms												
Symptoms are better or entirely gone	30 (32.6)	62 (67.4)	Ref		61 (58.1)	44 (41.9)	Ref		92 (82.1)	20 (17.9)	Ref	
Symptoms are the same or worse than before	4 (14.8)	23 (85.2)	2.78 (.88–8.77)	.08	1 (8.3)	11 (91.7)	4.81 (1.90–122.49)	.01	5 (100)	0 (0.0)	NE	.59
Symptomatology												
Fever and antipyretics use (last 24 h)												
No fever, without antipyretics use	22 (40.0)	33 (60.0)	Ref		48 (60.8)	31 (39.2)	Ref		83 (87.4)	12 (12.6)	Ref	
No fever, with antipyretics use	9 (16.4)	46 (83.6)	3.41 (1.39–8.34)	.007	12 (34.3)	23 (65.7)	2.97 (1.29–6.82)	.01	13 (61.9)	8 (38.1)	4.26 (1.46–12.39)	.008
Fever	3 (33.3)	6 (66.7)	1.33 (.30–5.90)	.71	2 (66.7)	1 (33.3)	0.77 (.07–8.91)	.84	1 (100)	0 (0.0)	NE	1.000
Presence of any symptom (last 48 h)	22 (24.2)	69 (75.8)	2.35 (.97–5.72)	.06	38 (48.7)	40 (51.3)	1.68 (.77–3.69)	.19	52 (78.8)	14 (21.2)	2.02 (.72–5.69)	.18
Median number of symptoms (IQR)	3 (42.9)	4 (57.1)	NA	.14	2 (40)	3 (60)	NA	.07	1 (33.3)	2 (66.7)	NA	.41

Abbreviations: Ct, cycle threshold value; IQR, interquartile range; NA, not applicable; NE, no estimate could be calculated due to perfect correlation; RADT, rapid antigen detection test; Ref, reference category; RT-PCR, real-time polymerase chain reaction.

^a^Regardless of timing of last vaccine dose.

^b^Among 121 participants with data on infectivity on day 5, 2 had missing information for RADT result and symptoms and 1 had missing information on RT-PCR CT result; among 120 participants with data on infectivity on day 7, 3 had missing information for RADT result and symptoms; among 121 participants with data on infectivity on day 10, 4 had missing information for RADT result and symptoms, and 1 had missing information on RT-PCR Ct result.

^c^Means were compared using student's t-test, proportions were compared using χ^2^ or Fisher exact test when appropriate.

^d^“Very mild” defined as able to carry out regular activities of daily living; “mild” defined as unable to carry out regular activities of daily living.

In terms of lineage, the BA.1 lineage was associated with a higher likelihood of infectivity on each follow-up visit than the BA.2 (*P* ≤ .02), although no difference was detected between BA.2 and BA.4/5.

From a clinical perspective, a lack of symptom improvement was predictive of ongoing infectivity on day 7 (OR, 4.81; *P* = .01) but not on day 5 or 10. Also, when compared to afebrile individuals who were not using antipyretics, those who were still using antipyretics were more likely to be infective at each follow-up visit (range of OR, 2.97 to 4.26; *P* ≤ .01 for each comparison).

**Figure 2.**
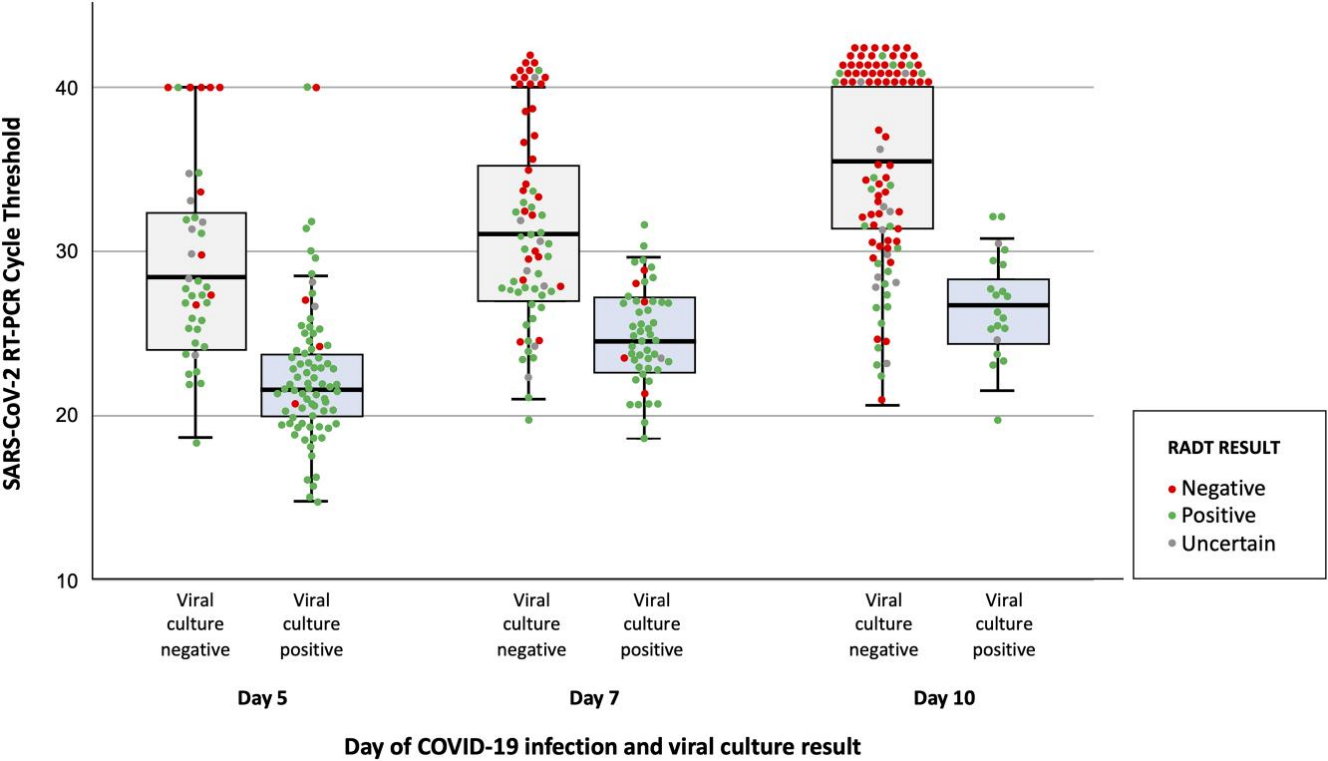
Box plot with overlaid jitter plot comparing SARS-CoV-2 RT-PCR Ct, RADT result, and viral culture positivity at day 5, 7, and 10 of COVID-19 among 121 healthcare workers. The horizontal line in each box indicates the median, whereas the top and bottom of the boxes represent the 75th and 25th percentile, respectively. Error bars represent 95% confidence intervals. Negative RT-PCR results were attributed a Ct value of 40 to facilitate data visualization. Abbreviations: COVID-19, coronavirus disease 2019; Ct, cycle threshold; RADT, rapid antigen diagnostic test; RT-PCR, real-time polymerase chain reaction; SARS-CoV-2, severe acute respiratory syndrome coronavirus 2.

**Figure 3.**
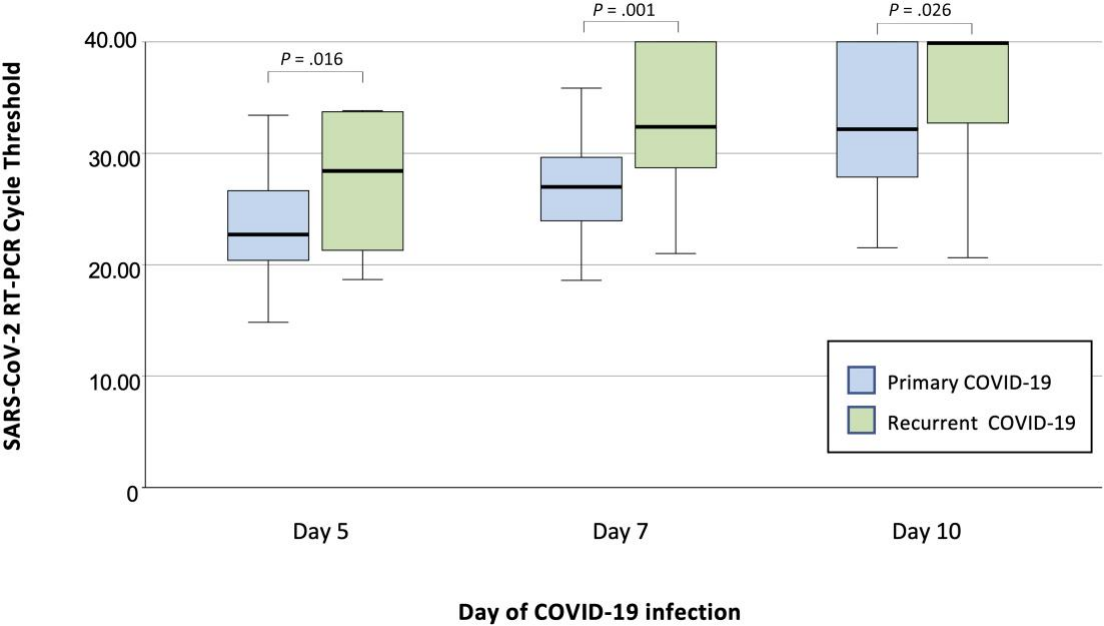
Box plot comparing SARS-CoV-2 RT-PCR Ct at day 5, 7, and 10 of primary versus recurrent COVID-19 infection. The horizontal line in each box indicates the median, whereas the top and bottom lines represent the 75th and 25th percentile, respectively. Error bars represent 95% confidence intervals. Negative RT-PCR results were attributed a Ct value of 40 to facilitate data visualization. Comparison between primary versus recurrent infections assessed by Mann-Whitney *U* test. Abbreviations: COVID-19, coronavirus disease 2019; Ct, cycle threshold; RADT, rapid antigen diagnostic test; RT-PCR, real-time polymerase chain reaction; SARS-CoV-2, severe acute respiratory syndrome coronavirus 2.

From the perspective of laboratory assays, a positive RADT result was associated with a higher likelihood of infectivity at day 5 (OR, 6.16; *P* = .004) and day 7 (OR, 10.93; *P* < .001). A positive RT-PCR was also significantly associated with infectivity at each follow-up, and there was an inverse association between the RT-PCR Ct value and ongoing infectivity (Figure 2). Notably, test results of participants with recurrent COVID-19 differed from those with primary infection. At each visit, they had significantly higher RT-PCR Ct values (Figure 3) and were significantly more likely to have a negative RADT test result (Table 3).

**Table 3.** Comparison of Rapid Antigen Detection Test Results of Healthcare Workers With Primary Versus Recurrent COVID-19

	Day 5 of Infection			Day 7 of Infection			Day 10 of Infection		
	Primary COVID-19 N (%)	Recurrent COVID-19 N (%)	*P* Value	Primary COVID-19 N (%)	Recurrent COVID-19 N (%)	*P* Value	Primary COVID-19 N (%)	Recurrent COVID-19 N (%)	*P* Value
RADT result (n)	100	20		99	19		98	19	
Positive RADT	86 (86.0)	11 (57.9)	.005	73 (73.7)	3 (15.8)	<.001	40 (40.8)	0 (.0)	<.001
Negative RADT	7 (7.0)	6 (31.6)		18 (18.2)	16 (84.2)		45 (45.9)	19 (100)	
Uncertain RADT	7 (7.0)	2 (10.5)		8 (8.1)	0 (0.0)		13 (13.3)	0 (0.0)	

Abbreviations: COVID-19, coronavirus disease 2019; RADT, rapid antigen detection test.

In multivariate analysis (Table 4), the following variables were independently associated ongoing infectivity: A RT-PCR Ct value <23 was associated with an increased probability of infectivity on each visit (adjusted OR [aOR] on day 5, 22.75; *P* < .001; aOR on day 7, 182.30; *P* < .001; and aOR on day 10; 24.71; *P* = .02). A Ct value ranging between 23 and 27 was also predictive of ongoing infectivity at day 7 and day 10. A history of previous COVID-19 was independently associated with a decreased probability of infectivity on day 5 (aOR, 0.005; *P* < .001). By contrast, there was no significant association between ongoing infectivity and the absence of fever (regardless of the use of antipyretics), symptom improvement, or RADT results.

**Table 4.** Predictors of Infectivity Among HCWs With COVID-19 (Multivariate Analysis)

	Day 5 (n = 121)			Day 7 (n = 117)			Day 10 (n = 117)		
	Adjusted OR	95% CI	*P* Value	Adjusted OR	95% CI	*P* Value	Adjusted OR	95% CI	*P* Value
Female sex	0.42	.09–2.06	.287	1.28	.31–5.34	.73	0.83	.16–4.18	.82
Age (y)									
20–39	Ref	…		Ref	…		Ref	…	
40–59	0.50	.15–1.68	.26	1.43	.47–4.34	.52	1.28	.36–4.63	.70
60–77	0.17	.02–1.63	.12	0.52	.06–4.71	.56	2.54	.25–26.31	.43
Immunity status stratified by timing of last vaccine and previous COVID-19									
No previous infection & last vaccine dose ≥6 m ago	Ref	…		Ref	…		Ref	…	
No previous infection & last vaccine dose <6 m ago	0.27	.03–2.33	.23	7.50	.89–62.83	.06	1.41	.14–14.15	.77
Previous infection, last vaccine dose> or <6 m ago^a^	0.005	.002–.16	.003	0.14	.003–6.61	.32	NE	…	
RADT result									
Negative	Ref	…		Ref	…		NE	…	
Positive	0.69	.11–4.43	.70	3.20	.74–13.91	.12	NE	…	
Uncertain	0.14	.1–1.48	.10	0.07	.002–1.82	.11	NE	…	
SARS-CoV-2 RT-PCR Ct									
≥27 (including negative)	Ref	…		Ref	…		Ref	…	
23–<27	1.30	.29–5.62	.73	4.81	1.52–15.25	.008	12.39	3.32–46.20	<.001
14–<23	22.75	3.89–133.05	<.001	182.30	8.83–3764.36	.001	24.71	1.53–398.50	.02
SARS-CoV-2 lineage^b^									
BA.1, BA.2 and subvariants	Ref	…		Ref	…		Ref	…	
BA.4, BA.5, BQ.1, XBB and subvariants	4.14	.50–33.97	.19	3.13	.46–21.43	.24	2.95	.52–16.70	.22
Evolution of symptoms									
Symptoms are better or entirely gone	Ref	…		Ref	…		NE	…	
Symptoms are the same or worse than before	0.52	.11–2.57	.42	18.67	.98–355.49	.05	NE	…	
Fever and antipyretic use^c^									
No fever, without antipyretics use	Ref	…		Ref	…		Ref	…	
No fever, with antipyretics use	4.83	1.30–17.98	.85	1.32	.40–4.35	.65	4.16	1.00–16.95	.047
Fever	1.21	.18–8.17	.85	NA	…		NA	…	

Abbreviations: CI, confidence interval; COVID-19, coronavirus disease 2019; Ct, cycle threshold; HCW, healthcare workers; NA, not applicable; NE, not estimable; OR, odds ratio; RADT, rapid antigen detection test; Ref, reference category; RT-PCR, real-time polymerase chain reaction; SARS-CoV-2, severe acute respiratory syndrome coronavirus 2.

^a^Regardless of timing of last vaccine dose.

^b^For 9 individuals with missing information, lineage BA.1/BA.2 or lineage BA.4/BA.5/BQ.1/XBB were assigned based on circulating variants at the date of testing.

^c^For the analyses of day 7 and day 10, “fever” and “no fever, with antipyretic use” were considered a single category.

### Performance of Return-to-Work Algorithms

**Figure 4.**
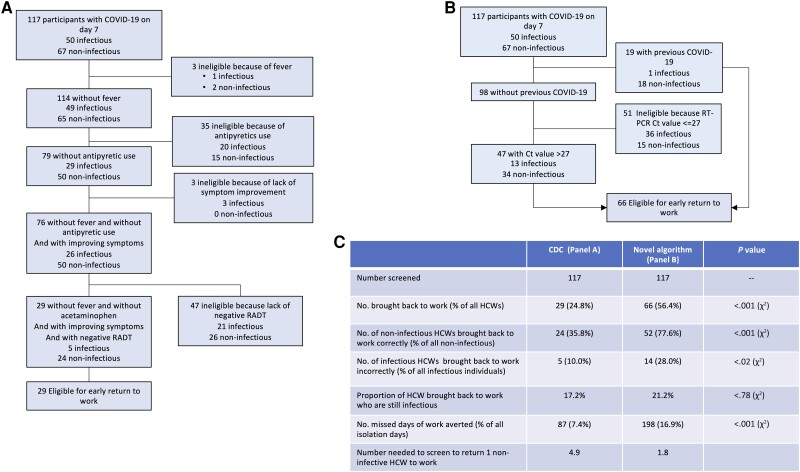
Performance of return-to-work criteria for healthcare workers with COVID-19. Panel *A* shows the performance of the US CDC Return to Work criteria on a cohort of healthcare workers with COVID-19. Panel *B* shows the performance of an alternative set of criteria derived from the current study. Panel *C* compares the CDC and alternative criteria. Abbreviations: CDC, Centers for Diseases Control and Prevention; COVID-19, coronavirus disease 2019; Ct, cycle threshold; HCW, healthcare workers; RADT, rapid antigen diagnostic test; RT-PCR, real-time polymerase chain reaction; SARS-CoV-2, severe acute respiratory syndrome coronavirus 2.

We applied the US CDC criteria to our cohort to identify non-infectious individuals on day 7 of COVID-19 (Figure 4) [7]. After exclusion of 4 participants with incomplete data, approximately three quarters (88/117; 75.2%) would be ineligible for return to work because of fever (n = 3), the use of antipyretics (n = 35), a lack of symptom improvement (n = 3), or a positive RADT (n = 47). Hence, only 29 HCWs (24.8%) would meet all the return-to-work criteria. Of these, 17.2% (5/29) were infectious by viral culture, and 82.8% (24/29) were non-infectious. Hence, this algorithm could identify a third (35.8%; 24/67) of all non-infectious individuals on day 7. If all 29 HCWs who fulfilled all criteria returned to work on day 7, this algorithm would decrease absenteeism by 7.4%.

We applied an alternative algorithm that used a history of previous COVID-19 and a RT-PCR Ct value >27 to predict loss of infectivity on day 7. This algorithm would identify 56.4% (66/117) of all HCWs as eligible for return to work and could avoid 198 days of absence (16.9%). Of these, 52 (78.8%) were non-infectious, and 14 (21.2%) were infectious. This algorithm would identify a greater proportion of all non-infectious HCWs than the CDC algorithm (77.6% vs 35.8%; *P* < .001). Even though it would return to work a greater number of infectious individuals, it would not significantly increase in the probability of returning to work an infectious HCW (21.2% vs 17.2% of all eligible HCWs, *P* = .78).

Given that approximately two thirds of individuals with recurrent COVID-19 were non-infectious by day 5, we explored various criteria that could accelerate their return-to-work (Supplementary Figure 1). Among these, a RT-PCR Ct value >27 could identify most (77%; 10/13) non-infectious individuals on day 5 with low probability (9%; 1/11) of returning to work an infectious HCW.

## DISCUSSION

The absence of healthcare workers with COVID-19 can exacerbate staff shortages [22]. There is a need to develop strategies to prevent unwarranted prolongation of absence. Most published studies on this topic were relatively small, limiting the identification of predictors of loss of infectivity [5, 23, 24].

In this prospective study, approximately three quarters (71.9%) were still shedding infectious viral particles on fifth day of infection, half (46.7%) on the seventh day, and a fifth (18.2%) on the tenth day. These results, along with other recent publications [25–28], differ from those of earlier studies that estimated the duration of infectivity to 10 days or less [5, 29]. A study of 66 individuals infected with the Omicron variant BA.1 reported that a quarter of participants were still infective on the tenth day of infection [9]. In another study, 8.5% were still shedding viable virus on day 14 [24].

Our study identifies important nuances regarding durations of infectivity. Nowadays, an increasing proportion of infections occurs in individuals who have hybrid immunity due to vaccination and previous COVID-19. Our study identified that vaccinated individuals with recurrent COVID-19 have a significantly shorter duration of infectivity as well as a distinct viral kinetic as evidenced by lower viral loads and earlier negativisation of RADT. A prepublication study of 1400 professional athletes also reported faster viral clearance by RT-PCR in individuals with recurrent COVID-19 compared with primary infections (4.9 vs 7.2 days, respectively) [30]. A cohort study in Alaska reported that individuals with previous COVID-19 infections were less likely to have a positive RADT result by day 5 compared to those with primary COVID-19 [25]. A similar phenomenon has been described with other *Coronaviridae*. A study performed three decades ago among volunteers infected with coronavirus 229E determined that reinfections led to a shorter duration of virus shedding compared with the primary episode [31]. Taken together, these findings profoundly alter our understanding of the infectivity and viral kinetic of recurrent COVID-19. We hypothesize these observations are due to the more diverse immune response to multiple antigenic sites on the virus reported with hybrid immunity compared with only vaccine-generated immunity [32].

Our study also identified that a higher RT-PCR Ct value was the strongest independent predictor of loss of infectivity. This confirms findings from other studies that demonstrated that higher Ct values correspond with a non-replicative virus [3,6, 26, 33–37]. Hence, RT-PCR Ct values could help determine the timing of return to work of HCWs. A negative RADT result was also predictive of loss of infectivity by bivariate analysis [38]. However, our multivariate analysis indicates that RT-PCR Ct values hold superior predictive capacity.

Our study also suggests that the guidance provided by the CDC to accelerate the return to work of infected HCWs is relatively stringent as it allows the return to work of only a third of all non-infectious individuals [7, 8]. Also, they appear to have moderate discriminatory power as up to a sixth of those eligible for return to work are still shedding viable virus. By contrast, an alternative algorithm using RT-PCR Ct values and a history of previous COVID-19 could be able to return to work a greater proportion of HCWs on day 7 without significantly increasing the probability of returning to work an infectious HCW. Importantly, the performance of these algorithms will be influenced by whether the COVID-19 episode is a first episode or a recurrence. In our opinion, such distinction will be essential when updating these algorithms.

Our study has strengths. To our knowledge, this study is the first to demonstrate that individuals with recurrent COVID-19 have significantly shorter durations of infectivity using the gold standard of viral culture. It is among the largest that assessed infectivity using viral culture. Our laboratory technique was sensitive and could detect viable viruses in many individuals 10 days into their infection. It also has limitations. The study enrolled mainly young, healthy, and highly immunized female participants with mild COVID-19, so generalizability to other populations is uncertain. There are limitations to using cycle threshold values to predict infectivity as they cannot be compared across testing platforms, and regulation often prevents the release of these results [39]. The correlation between culture positivity and risk of transmission remains unclear [9]. Additional studies, including validation with an external cohort, would be required to better inform return-to-work policies [20, 21].

In conclusion, our study detected a higher RT-PCR Ct value and COVID-19 reinfection as independent predictors of loss of infectivity in a highly vaccinated population, and suggests that return-to-work algorithms could be optimized to limit absenteeism [10]. Further studies are needed to further characterize the viral kinetics of COVID-19 reinfections as they appear to differ from those with primary COVID-19.

## Supplementary Data

Supplementary materials are available at *Clinical Infectious Diseases* online. Consisting of data provided by the authors to benefit the reader, the posted materials are not copyedited and are the sole responsibility of the authors, so questions or comments should be addressed to the corresponding author.

## Supplementary Material

ciad535_Supplementary_Data

## Notes

***Author Contributions.*** Concept and Design: S. D., J. F., H. C., G. D. S., J. L., J. V., Y. L. Acquisition, analysis, or interpretation of data: S. D., T. R., H. C., J. F., I. L., S. N. B., L. P., Y. L. Drafting of manuscript: S. D., Y. L. Critical revision of the manuscript for important intellectual content: H. C., T. R., J. F., S. C.o, I. L., J. L., L. P., S. N. B., J. V., P. S., J. C., G. D. S. Statistical analyses: S. C., G. D. S., Y. L.. Obtained funding: Y. L., G. D. S. Administrative, technical or material support: S. D., Y. L.. Supervision: Y. L., G. D. S.

***Data availability statement.*** Y. L. had full access to all the data in the study and takes responsibility for the integrity of the data and the accuracy of the data analysis. Data not publicly available.

***Financial support*.** This work was supported by the Ministère de la Santé et des Services Sociaux (MSSS) du Québec. The funding source had no role in the design of the study, execution, analyses, interpretation of data, writing of report or decision to submit the manuscript. J. F. reports that sequencing was funded by MSSS and Public Health Agency of Canada (PHAC), and Reagents and staff was paid by a grant from MSSS. Some reagants were provided by PHAC.

## References

[1] Helmy YA, Fawzy M, Elaswad A, Sobieh A, Kenney SP, Shehata AA. The COVID-19 pandemic: a comprehensive review of taxonomy, genetics, epidemiology, diagnosis, treatment, and control. J Clin Med 2020; 9:1225. 32344679 10.3390/jcm9041225 PMC7230578

[2] Kadire SR, Fabre V, Wenzel RP. Doctor, how long should I isolate? N Engl J Med 2021; 384:e47. 33691057 10.1056/NEJMclde2100910

[3] Arons MM, Hatfield KM, Reddy SC, et al Presymptomatic SARS-CoV-2 infections and transmission in a skilled nursing facility. N Engl J Med 2020; 382:2081–90. 32329971 10.1056/NEJMoa2008457 PMC7200056

[4] Wolfel R, Corman VM, Guggemos W, et al Virological assessment of hospitalized patients with COVID-2019. Nature 2020; 581:465–469. 32235945 10.1038/s41586-020-2196-x

[5] Cevik M, Tate M, Lloyd O, Maraolo AE, Schafers J, Ho A. SARS-CoV-2, SARS-CoV, and MERS-CoV viral load dynamics, duration of viral shedding, and infectiousness: a systematic review and meta-analysis. Lancet Microbe 2021; 2:e13–22. 33521734 10.1016/S2666-5247(20)30172-5 PMC7837230

[6] Bullard J, Dust K, Funk D, et al Predicting infectious severe acute respiratory syndrome coronavirus 2 from diagnostic samples. Clin Infect Dis 2020; 71:2663–6. 32442256 10.1093/cid/ciaa638 PMC7314198

[7] US Centers for Diseases Control and Prevention . Interim guidance for managing healthcare personnel with SARS-CoV-2 infection or exposure to SARS-CoV-2 (2022). Available at: https://www.cdc.gov/coronavirus/2019-ncov/hcp/guidance-risk-assesment-hcp.html. Accessed 10 May 2023.

[8] European Center for Disease Control and Prevention . Guidance on quarantine of close contacts to COVID-19 cases and isolation of COVID-19 cases, in the current epidemiological situation, 7 January 2022. Available at: https://www.ecdc.europa.eu/en/covid-19/prevention-and-control/quarantine-and-isolation. Accessed 10 May 2023.

[9] Boucau J, Marino C, Regan J, et al Duration of shedding of culturable virus in SARS-CoV-2 Omicron (BA.1) infection. N Engl J Med 2022; 387:275–7. 35767428 10.1056/NEJMc2202092 PMC9258747

[10] Black JRM, Bailey C, Przewrocka J, Dijkstra KK, Swanton C. COVID-19: the case for health-care worker screening to prevent hospital transmission. Lancet 2020; 395:1418–20. 32305073 10.1016/S0140-6736(20)30917-X PMC7162624

[11] World Health Organization . WHO R&D blueprint novel coronavirus COVID-19 therapeutic trial synopsis. 2020. Available at: https://cdn.who.int/media/docs/default-source/blue-print/covid-19-therapeutic-trial-synopsis.pdf? sfvrsn=44b83344_1&download=true. Accessed 18 February 2020.

[12] von Elm E, Altman DG, Egger M, et al The strengthening the reporting of observational studies in epidemiology (STROBE) statement: guidelines for reporting observational studies. J Clin Epidemiol 2008; 61:344–9. 18313558 10.1016/j.jclinepi.2007.11.008

[13] Folgueira MD, Luczkowiak J, Lasala F, Perez-Rivilla A, Delgado R. Prolonged SARS-CoV-2 cell culture replication in respiratory samples from patients with severe COVID-19. Clin Microbiol Infect 2021; 27:886–91. 33631334 10.1016/j.cmi.2021.02.014 PMC7898982

[14] Longtin Y, Charest H, Quach C, et al Infectivity of healthcare workers diagnosed with coronavirus disease 2019 (COVID-19) approximately 2 weeks after onset of symptoms: a cross-sectional study. Infect Control Hosp Epidemiol 2021; 43:1–3. 10.1017/ice.2020.1420 PMC7853751 33427133

[15] Wurtz N, Penant G, Jardot P, Duclos N, La Scola B. Culture of SARS-CoV-2 in a panel of laboratory cell lines, permissivity, and differences in growth profile. Eur J Clin Microbiol Infect Dis 2021; 40:477–84. 33389257 10.1007/s10096-020-04106-0 PMC7778494

[16] CoV Sequencing Pipeline . Available at: https://genpipes.readthedocs.io/en/latest/user_guide/pipelines/gp_covseq.html, 2019. Accessed 10 May 2023.

[17] O'Toole A, Scher E, Underwood A, et al Assignment of epidemiological lineages in an emerging pandemic using the pangolin tool. Virus Evol 2021; 7:veab064. 34527285 10.1093/ve/veab064 PMC8344591

[18] Papenburg J, Campbell JR, Caya C, et al Adequacy of serial self-performed SARS-CoV-2 rapid antigen detection testing for longitudinal mass screening in the workplace. JAMA Netw Open 2022; 5:e2210559. 35522284 10.1001/jamanetworkopen.2022.10559 PMC9077488

[19] Killingley B, Mann AJ, Kalinova M, et al Safety, tolerability and viral kinetics during SARS-CoV-2 human challenge in young adults. Nat Med 2022; 28:1031–41. 35361992 10.1038/s41591-022-01780-9

[20] Bender R, Lange S. Adjusting for multiple testing–when and how? J Clin Epidemiol 2001; 54:343–9. 11297884 10.1016/s0895-4356(00)00314-0

[21] Rothman KJ . No adjustments are needed for multiple comparisons. Epidemiology 1990; 1:43–6. 2081237

[22] Poon YR, Lin YP, Griffiths P, Yong KK, Seah B, Liaw SY. A global overview of healthcare workers’ turnover intention amid COVID-19 pandemic: a systematic review with future directions. Hum Resour Health 2022; 20:70. 36153534 10.1186/s12960-022-00764-7 PMC9509627

[23] Wu Y, Guo Z, Yuan J, et al Duration of viable virus shedding and polymerase chain reaction positivity of the SARS-CoV-2 Omicron variant in the upper respiratory tract: a systematic review and meta-analysis. Int J Infect Dis 2023; 129:228–35. 36804640 10.1016/j.ijid.2023.02.011 PMC9937726

[24] Keske S, Guney-Esken G, Vatansever C, et al Duration of infectious shedding of SARS-CoV-2 Omicron variant and its relation with symptoms. Clin Microbiol Infect 2023; 29:221–4. 35853589 10.1016/j.cmi.2022.07.009 PMC9287585

[25] Lefferts B, Blake I, Bruden D, et al Antigen test positivity after COVID-19 isolation—Yukon-Kuskokwim delta region, Alaska, January–February 2022. MMWR Morb Mortal Wkly Rep 2022; 71:293–8. 35202352 10.15585/mmwr.mm7108a3

[26] Qi L, Yang Y, Jiang D, et al Factors associated with the duration of viral shedding in adults with COVID-19 outside of Wuhan, China: a retrospective cohort study. Int J Infect Dis 2020; 96:531–7. 32425636 10.1016/j.ijid.2020.05.045 PMC7231495

[27] Landon E, Bartlett AH, Marrs R, Guenette C, Weber SG, Mina MJ. High rates of rapid antigen test positivity after 5 days of isolation for COVID-19. medRxiv 2022. doi:10.1101/2022.02.01.22269931

[28] Stiefel U, Bhullar D, Zabarsky TF, et al Healthcare personnel frequently have positive severe acute respiratory syndrome coronavirus 2 (SARS-CoV-2) antigen tests 5 days or more after diagnosis of coronavirus disease 2019 (COVID-19). Infect Control Hosp Epidemiol 2022; 43:1985–7. 35131000 10.1017/ice.2022.21 PMC8861541

[29] Takahashi K, Ishikane M, Ujiie M, et al Duration of infectious virus shedding by SARS-CoV-2 Omicron variant-infected vaccinees. Emerg Infect Dis 2022; 28:998–1001. 35290176 10.3201/eid2805.220197 PMC9045443

[30] Kissler SM, Hay JA, Fauver JR, et al Viral kinetics of sequential SARS-CoV-2 infections. *medRxiv* 2023:2023.03.03.23286775 [Preprint]. March 06, 2023. Available from: 10.1101/2023.03.03.23286775 PMC10556125 37798265

[31] Callow KA, Parry HF, Sergeant M, Tyrrell DA. The time course of the immune response to experimental coronavirus infection of man. Epidemiol Infect 1990; 105:435–46. 2170159 10.1017/s0950268800048019 PMC2271881

[32] Bobrovitz N, Ware H, Ma X, et al Protective effectiveness of previous SARS-CoV-2 infection and hybrid immunity against the Omicron variant and severe disease: a systematic review and meta-regression. Lancet Infect Dis 2023; 23:556–67. 36681084 10.1016/S1473-3099(22)00801-5 PMC10014083

[33] Gniazdowski V, Paul Morris C, Wohl S, et al Repeated coronavirus disease 2019 molecular testing: correlation of severe acute respiratory syndrome coronavirus 2 culture with molecular assays and cycle thresholds. Clin Infect Dis 2021; 73:e860–9. 33104776 10.1093/cid/ciaa1616 PMC7665437

[34] Kim MC, Cui C, Shin KR, et al Duration of culturable SARS-CoV-2 in hospitalized patients with Covid-19. N Engl J Med 2021; 384:671–3. 33503337 10.1056/NEJMc2027040 PMC7934323

[35] Longtin Y, Parkes LO, Charest H, et al Persistence of infectivity in elderly individuals diagnosed with severe acute respiratory coronavirus virus 2 (SARS-CoV-2) infection 10 days after onset of symptoms: a cross-sectional study. Infect Control Hosp Epidemiol 2021; 44:659–62. 34866565 10.1017/ice.2021.502 PMC8692849

[36] Aranha C, Patel V, Bhor V, Gogoi D. Cycle threshold values in RT-PCR to determine dynamics of SARS-CoV-2 viral load: an approach to reduce the isolation period for COVID-19 patients. J Med Virol 2021; 93:6794–7. 34264527 10.1002/jmv.27206 PMC8426941

[37] Jefferson T, Spencer EA, Brassey J, Heneghan C. Viral cultures for COVID-19 infectious potential assessment—a systematic review. Clin Infect Dis 2020; 73:e3884–99. 10.1093/cid/ciaa1764 PMC7799320 33270107

[38] Bouton TC, Atarere J, Turcinovic J, et al Viral dynamics of omicron and delta severe acute respiratory syndrome coronavirus 2 (SARS-CoV-2) variants with implications for timing of release from isolation: a longitudinal cohort study. Clin Infect Dis 2023; 76:e227–33. 35737948 10.1093/cid/ciac510 PMC9278204

[39] Binnicker MJ . Can testing predict SARS-CoV-2 infectivity? The potential for certain methods to be surrogates for replication-competent virus. J Clin Microbiol 2021; 59:e0046921. 34346713 10.1128/JCM.00469-21 PMC8525553

