# Supplementary material for: Timing and Predictors of Loss of Infectivity Among Healthcare Workers With Mild Primary and Recurrent COVID-19: A Prospective Observational Cohort Study
Source: Clin Infect Dis. 2023 Sep 7;78(3):613–24. doi: 10.1093/cid/ciad535 (PMC10954326; doi:10.1093/cid/ciad535)
Supplement: ciad535_Supplementary_Data [file ciad535_supplementary_data.pdf]

Supplemental Online Content

Dzieciolowska S, Charest H, Roy T et al. Timing and Predictors of Loss of Infectivity among Healthcare Workers with Mild Primary and Recurrent COVID-19: a Prospective Observational Cohort Study

eMethods. Whole Genome Sequencing

eTable 1. Association between symptomatology and infectivity on day 5, 7 and 10 of COVID-19 among healthcare workers (bivariate analyses)

eFIGURE 1. Performance of return-to-work criteria for healthcare workers with recurrent COVID-19 on the fifth day of their infection

This supplemental material has been provided by the authors to give readers additional information about their work.

eMethods

Whole Genome Sequencing and Sequence Data Analysis

Nucleic acids were extracted from 0.2-mL aliquots using the NUCLISENS easyMAG (bioMérieux, Marcy-l'Étoile, France). The viral RNA was sequenced using Illumina technology, and a targeted SARS-CoV-2 amplification strategy was employed based on the ARTIC V4.1 primer scheme.[1] Libraries were prepared with the Illumina COVIDSeq Test kit according to manufacturer's recommendation[2] and sequenced with a NextSeq 1000 (Illumina). Data analysis was conducted using the GenPipes Covseq pipeline,[3] which performed alignment and produced variant calls. Initially, host reads were removed by aligning them to a hybrid reference consisting of human (GRCh38) and Wuhan-Hu-1 SARS-CoV-2 reference (MN908947.3) sequences. Raw reads were trimmed using cutadapt (v2.10), and then aligned to the reference using bwa-mem (v0.7.17).[4] The resulting aligned reads were filtered using sambamba (v0.7.0),[5] which removed paired reads with insert sizes outside the 60-300 bp range, unmapped reads, and all secondary alignments. Remaining ARTIC primers (v4.1) were trimmed using iVar (v1.3).[6] To generate a consensus sequence, a pileup was produced using Samtools (v1.12),[7] which was then used as input for FreeBayes (v1.3.4) to create a consensus sequence for regions with a minimum of 10× depth and using reads with a Q score > 20.[8] Mutations were annotated with snpEff (v4.5).[9] Single nucleotide variants below 5% allele frequency were filtered out. A full description of the process can be found here:

https://c3g.github.io/covseq_McGill/SARS_CoV2_Sequencing/Illumina_overview.html.

Variant identification and detection of recombination

Variant identification was performed using the Pangolin program (v4.2, UShER analysis mode),[10] and the program ncov-recombinant (v.0.6.0) was used to characterize recombinant lineages non-identified by Pangolin.[11]

eMethods References

1. Initial implementation of an ARTIC bioinformatics platform for nanopore sequencing of nCoV2019 novel coronavirus. https://github.com/artic-network/artic-ncov2019, Accessed March 14, 2023.

2. Illumina COVIDSeq Test Instructions for Use. https://support.illumina.com/downloads/illumina-covidseq-test-instructions-for-use-1000000128490.html. Accessed March 14, 2023.

3. CoV Sequencing Pipeline. https://genpipes.readthedocs.io/en/genpipes-v4.1.2/user_guide/pipelines/gp_covseq.html, 2019.

4. Li H, Durbin R. Fast and accurate short read alignment with Burrows-Wheeler transform. *Bioinformatics*. Jul 15 2009;25(14):1754-60. doi:10.1093/bioinformatics/btp324

5. Tarasov A, Vilella AJ, Cuppen E, Nijman IJ, Prins P. Sambamba: fast processing of NGS alignment formats. *Bioinformatics*. Jun 15 2015;31(12):2032-4. doi:10.1093/bioinformatics/btv098

6. Castellano S, Cestari F, Faglioni G, et al. iVar, an Interpretation-Oriented Tool to Manage the Update and Revision of Variant Annotation and Classification. *Genes (Basel)*. Mar 8 2021;12(3)doi:10.3390/genes12030384

7. Li H, Handsaker B, Wysoker A, et al. The Sequence Alignment/Map format and SAMtools. *Bioinformatics*. Aug 15 2009;25(16):2078-9. doi:10.1093/bioinformatics/btp352

8. Garrison E, Marth G. Haplotype-based variant detection from short-read sequencing. *arXiv preprint arXiv:12073907*. 2012;

9. Cingolani P, Platts A, Wang le L, et al. A program for annotating and predicting the effects of single nucleotide polymorphisms, SnpEff: SNPs in the genome of Drosophila melanogaster strain w1118; iso-2; iso-3. *Fly (Austin)*. Apr-Jun 2012;6(2):80-92. doi:10.4161/fly.19695

10. O'Toole A, Scher E, Underwood A, et al. Assignment of epidemiological lineages in an emerging pandemic using the pangolin tool. *Virus Evol*. 2021;7(2):veab064. doi:10.1093/ve/veab064

11. ktmeaton/ncov-recombinant. https://github.com/ktmeaton/ncov-recombinant, 2023.

eTable 1. Association between symptomatology and infectivity on day 5, 7 and 10 of COVID-19 among healthcare workers (bivariate analyses)

Explanatory variable	Day 5				Day 7				Day 10			
	Absence of infectivity n (line %)	Presence of infectivity n (line %)	OR (95% CI)	P-value[a]	Absence of infectivity n (line %)	Presence of infectivity n (line %)	OR (95% CI)	P-value[a]	Absence of infectivity n (line %)	Presence of infectivity n (line %)	OR (95% CI)	P-value[a]
**Symptoms**												
Chills	1 (6.7)	14 (93.3)	6.51 (0.8251.59)	0.08	2 (28.6)	5 (71.4)	3.00 (0.56-16.13)	0.20	3 (75.0)	1 (25.0)	1.65 (0.16-16.72)	0.67
Cough	20 (25.3)	59 (74.7)	1.58 (0.70-3.62)	0.27	31 (46.3)	36 (53.7)	1.89 (0.90-4.00)	0.09	41 (75.9)	13 (24.1)	2.54 (0.93-6.92)	0.07
Fatigue	12 (20.3)	47 (79.7)	2.27 (1.00-5.16)	0.051	22 (50.0)	22 (50.0)	1.21 (0.57-2.56)	0.62	27 (75.0)	9 (25.0)	2.12 (0.79-5.69)	0.14
Myalgia	7 (21.2)	26 (78.8)	1.70 (0.66-4.40)	0.27	6 (33.3)	12 (66.7)	2.60 (0.90-7.50)	0.08	9 (81.8)	2 (18.2)	1.09 (0.22-5.46)	0.92
Sore throat	9 (18.8)	39 (81.3)	2.36 (0.98-5.64)	0.06	16 (50.0)	16 (50.0)	1.18 (0.52-2.66)	0.69	18 (94.7)	1 (5.3)	0.23 (0.03-1.84)	0.17
Headache	10 (22.7)	34 (77.3)	1.60 (0.68-3.77)	0.28	10 (38.5)	16 (61.5)	2.13 (0.87-5.21)	0.10	14 (87.5)	2 (12.5)	0.66 (0.14-3.16)	0.60
Dizziness	4 (33.3)	8 (66.7)	0.78 (0.22-2.78)	0.70	5 (33.3)	10 (66.7)	2.53 (0.81-7.94)	0.11	10 (90.9)	1 (9.1)	0.46 (0.06-3.79)	0.47
Rhinorrhea and/or congestion	19 (23.2)	63 (76.8)	2.26 (0.98-5.20)	0.055	31 (47.7)	34 (52.3)	1.16 (0.77-3.38)	0.20	36 (76.6)	11 (23.4)	2.07 (0.78-5.48)	0.14
Diarrhea	4 (25.0)	12 (75.0)	1.23 (0.37-4.13)	0.73	4 (36.4)	7 (63.6)	2.11 (0.58-7.66)	0.25	2 (50.0)	2 (50.0)	5.28 (0.70-39.93)	0.11
Loss of appetite	8 (28.6)	20 (71.4)	1.00 (0.39-2.55)	1.00	7 (33.3)	14 (66.7)	2.68 (0.99-7.24)	0.052	11 (68.8)	5 (31.3)	2.61 (0.79-8.57)	0.12

Footnote : [a] Proportions were compared using chi-square or fisher exact test when appropriate

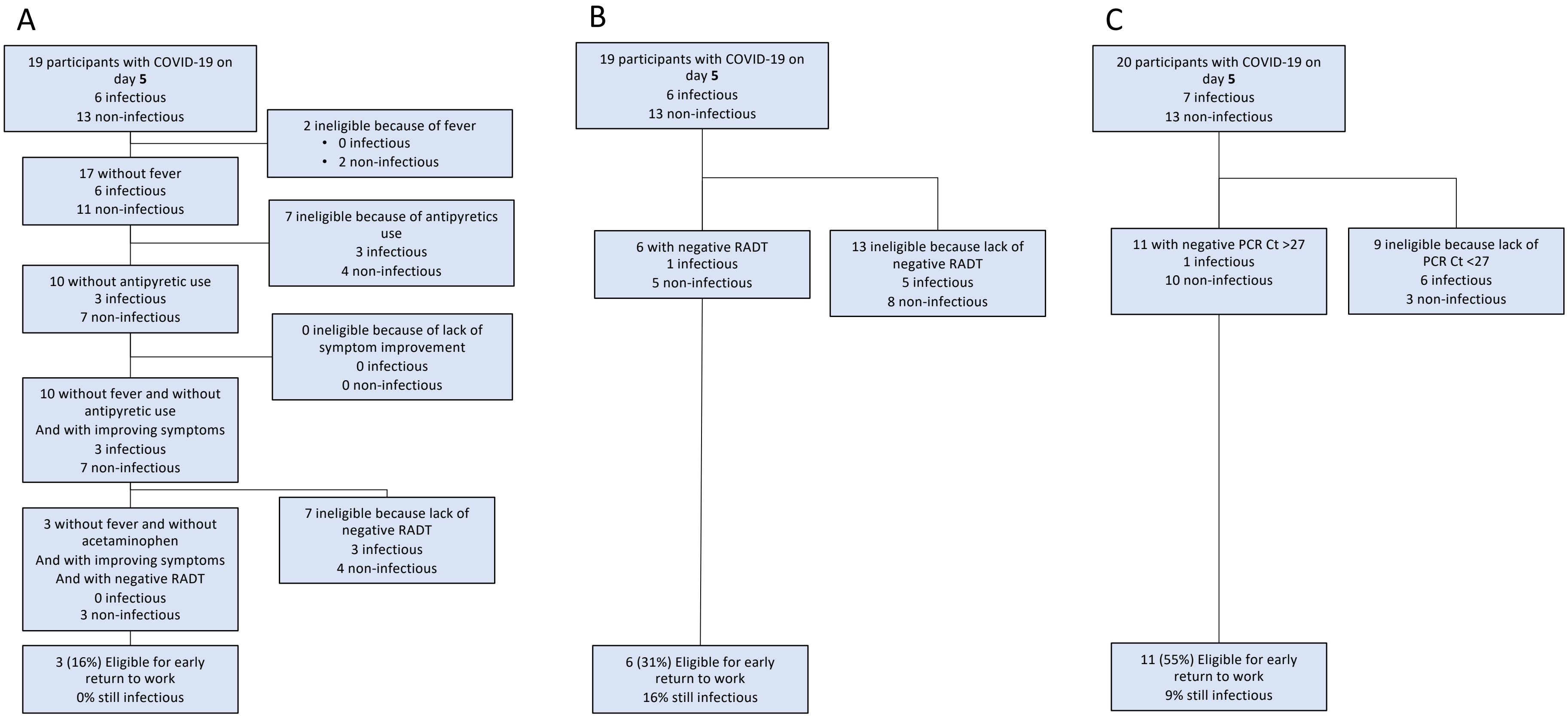

eFIGURE 1. Performance of return-to-work criteria for healthcare workers with recurrent COVID-19 on the fifth day of their infection. Panel A shows the performance of the Centers for Diseases Control and Prevention (US CDC) Return to Work criteria. **Panels B and C** shows the performance of alternate algorithms relying on rapid antigen detection tests (RADT) and RT-PCR cycle threshold (Ct) values.
